# Dopamine cross-sensitization between psychostimulant drugs and stress in healthy male volunteers

**DOI:** 10.1038/tp.2016.6

**Published:** 2016-02-23

**Authors:** L Booij, K Welfeld, M Leyton, A Dagher, I Boileau, I Sibon, G B Baker, M Diksic, J-P Soucy, J C Pruessner, E Cawley-Fiset, K F Casey, C Benkelfat

**Affiliations:** 1 Department of Psychology, Concordia University, Montreal, QC, Canada; 2 CHU Sainte Justine Hospital Research Center, University of Montreal, Montreal, QC, Canada; 3 Department of Psychiatry, McGill University, Montreal, QC, Canada; 4 Center for Studies in Behavioral Neurobiology, Concordia University, Montreal, QC, Canada; 5 McConnell Brain Imaging Centre, Montreal Neurological Institute, McGill University, Montreal, QC, Canada; 6 Center for Addiction and Mental Health, University of Toronto, Toronto, ON, Canada; 7 Pole de Neurosciences Cliniques, Hôpital Pellegrin, CHU Bordeaux, Bordeaux, France; 8 Neurobiology Research Unit, Department of Psychiatry, Institute of Neuroscience and Mental Health, University of Alberta, Edmonton, AB, Canada; 9 Douglas Mental Health University Institute, Department of Psychiatry, McGill University, Montreal, QC, Canada

## Abstract

Dysregulation of the stress response system is a potential etiological factor in the development of and relapse to multiple neuropsychiatric disorders. Previously we reported that repeated intermittent d-amphetamine administration can lead to progressively greater dopamine release, thereby providing evidence of drug-induced neurochemical sensitization. Here, we test the hypothesis that repeated exposure to d-amphetamine increases dopaminergic responses to stress; that is, produces cross-sensitization. Using positron emission tomography, we measured in 17 healthy male volunteers (mean±s.d.=22.1±3.4 years) [^11^C]raclopride binding responses to a validated psychosocial stress task before and 2 weeks after a regimen of repeated d-amphetamine (3 × 0.3 mg kg^−1^, by mouth; *n*=8) or placebo (3 × lactose, by mouth; *n*=9). Mood and physiological measurements were recorded throughout each session. Before the d-amphetamine regimen, exposure to the stress task increased behavioral and physiological indices of stress (anxiety, heart rate, cortisol, all *P*⩽0.05). Following the d-amphetamine regimen, the stress-induced cortisol responses were augmented (*P*<0.04), and voxel-based analyses showed larger stress-induced decreases in [^11^C]raclopride non-displaceable binding potential across the striatum. In the placebo group, re-exposure to stress led to smaller clusters of decreased [^11^C]raclopride binding, primarily in the sensorimotor striatum (*P*<0.05). Together, this study provides evidence for drug × stress cross-sensitization; moreover, random exposure to stimulants and/or stress cumulatively, while enhancing dopamine release in striatal areas, may contribute to a lowered set point for psychopathologies in which altered dopamine neurotransmission is invoked.

## Introduction

Stress is a key contributing factor in the development and exacerbation of chronic relapsing neuropsychiatric disorders, including addiction and psychosis. One potentially involved process is ‘sensitization' that is*,* following repeated exposure to stressors and/or psychostimulant drugs, some effects can become progressively greater.^1, 2, 3^ In susceptible individuals, these enhanced responses have been proposed to influence illness onset and relapse. ^4, 5, 6, 7^

In animals, ‘sensitization' to psychostimulants is subject to cross-sensitization with stress.^8, 9^ For instance, in rodents, repeated exposure to psychostimulants increases the ability of stressors to precipitate motor activity, drug self-administration and dopamine (DA) release.^10, 11^ Conversely, exposure to experimental stress can increase the behavioral and DA response to psychostimulants.^3, 12, 13, 14^ Although the neurobiological substrate mediating cross-sensitization between stress and psychostimulants is not fully understood, there is evidence that it includes the interaction between the hypothalamic–pituitary–adrenal axis and DA projections, in particular those arising from the mesencephalon.^15^ A number of studies showed that both stress and d-amphetamine activate the hypothalamic–pituitary–adrenal axis, resulting in increased cortisol levels.^16^ Glucocorticoids, in turn, may facilitate DA release through several mechanisms, including affecting tyrosine hydroxylase, monoamine oxidase-A and DA reuptake.^16^ Conceivably, this could lead to a greater DA response on stress exposure. Indeed, removal of the major source of endogenous glucocorticoids, through surgery or pharmacological blockade (metyrapone), diminishes the development of drug-induced DA sensitization.^17^

We have previously reported a persistent increase in DA release on stimulant re-exposure in healthy humans who had undergone a subchronic regimen of d-amphetamine (three doses within 1 week) when tested at least 2 weeks following the last stimulant dose, an observation interpreted as evidence of neurochemical sensitization.^18^ The present study follows up on this initial observation^18^ to test the hypothesis that the same d-amphetamine regimen would lead to a greater DA response to a psychosocial stressor administered 2 weeks following the last stimulant dose; that is, evidence of cross-sensitization.

## Materials and methods

### Participants

Healthy males were recruited through on-line advertisements in the university network and local newspapers. Following a telephone interview to assess initial eligibility, participants underwent a full in-lab screening including: (1) a semi-structured psychiatric interview (Structured Clinical Interview for DSM-IV: Patient Edition, SCID-NP),^19^ (2) a complete physical examination including laboratory testing and an electrocardiogram and (3) measures of self-esteem and trait anxiety, including a questionnaire of competence and control,^20^ the Rosenberg self-esteem scale^21^ and the State-Trait Anxiety Inventory.^22^ Main exclusion criteria included: (1) major medical /neurological illness or the use of medication likely to affect brain function or confound positron emission tomography (PET) results; (2) a personal or family history of Axis I disorders; (3) regular past or present drug use (that is, exposure to stimulant drugs or hallucinogens/sedatives in the past 12 months); (4) lifetime use of stimulants, sedatives or hallucinogens exceeding four exposures total; (5) frequent use of tobacco (⩾5 cigarettes per day); (6) frequent use of cannabis (greter than two uses per week); (7) testing positive on a urine toxicology screen for illicit drug abuse on the days of study (Triage-TM); and (8) meeting other PET/magnetic resonance imaging (MRI) exclusion criteria (see Supplementary Information). The study was approved by the Montreal Neurological Institute Research Ethics Board. All the participants provided written informed consent.

### Design overview

**Figure 1:**
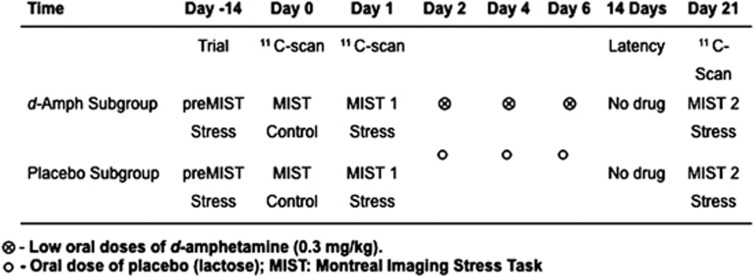
Experimental design of the study. PreMIST=practice session, before first PET [^11^C]raclopride scan. MIST control=PET [^11^C]raclopride scan in association with the low stress control task. MIST 1=PET [^11^C]raclopride scan with the stressful MIST task before the d-amphetamine or placebo regimen. MIST 2=PET [^11^C]raclopride scan with the stressful MIST task 14 days after the last drug (d-amphetamine or placebo) dose. PET, positron emission tomography.

Eligible participants received either d-amphetamine (0.3 mg kg^−1^, by mouth) or placebo on three separate days, every 48 h, in the same environment (on the PET gantry), following similar procedures and assessments as in our initial study.^18^ All the participants underwent three 60-minute PET [^11^C]raclopride scan (~7 mCi) sessions, during which they were exposed to the Montreal Imaging Stress Task (MIST; Figure 1). One PET [^11^C]raclopride scan was conducted with the control task (MIST control), whereas the two other PET [^11^C]raclopride scans were obtained with the MIST stress task to assess DA responses to stress before (MIST 1) and 14 days after the last drug (d-amphetamine or placebo) dose (MIST 2). All the participants underwent an anatomical high-resolution T1-weighted MRI scan for the purpose of PET registration. To minimize the influence of habituation to the MIST, participants performed the MIST task (pre MIST) once before the first PET session, as habituation of the stress response tends to be strongest between the first and second exposure to the same stress task.^23, 24^ Participants were asked to fast and abstain from caffeine and tobacco for a minimum of 4 h before each session. All seven sessions took place over a period of ~21 days, as described in Figure 1 (see Supplementary Information). They were instructed not to use any drugs throughout the entire study period. This was confirmed by a negative urine drug test at the beginning of each session.

### Experimental stress task

The MIST is a validated stress task based on the Trier Mental Challenge task^25^ and adapted for use in an imaging environment.^26^ We used three 12-min blocks, each with four 3-min segments. During the task, arithmetic tasks are presented in the scanner via a computer screen. Participants answered using a computer mouse. Task difficulty and time constraint for each calculation are adjusted automatically by the computer algorithm in real time depending on the participant's performance, so as to be slightly beyond each individual's capability. After each trial, the computer screen displays feedback about the participant's performance (correct, incorrect, timeout); following each segment, a negative feedback is provided in two complementary ways: by the program and by a confederate. Participants were led to believe that their performance was below expectations, and were asked to increase performance to meet requirements.

This task has been shown to elicit behavioral and hormonal responses to stress and has been associated with striatal DA release in healthy volunteers, including in its more ventral portion.^26, 27^ During the sensorimotor control condition (MIST control), participants performed simple arithmetic for 36 min, as described above, without time constraints, signs of visible progress, sound or negative feedback. Participants were debriefed at the end of the last PET stress session, and were told that the task was customized to be outside of their mental ability and was not meant to measure their arithmetic skills.

Subjective behavioral changes were assessed with the Profile of Mood States^28^ and the State-Trait Anxiety Inventory,^22^ before and immediately after the end of each MIST exposure; as well as at the end of the test session when outside the scanner (data not shown). Blood samples for cortisol and heart rate measures (MP100-Biopac Systems) were collected at baseline and every 12 min throughout each session (Figure 1).

### Voxel-wise parametric map and *t*-statistics

The PET images were corrected for between-frame motion artefacts^29^ and co-registered to each individual's MRI. The MRI and PET images were linearly transformed into stereotactic space using the Montreal Neurological Institute-305 template.^30^ [^11^C]Raclopride non-displaceable binding potential (BP_ND_=*f*_ND_*B*_avail_/K_D_) was estimated at each voxel, using a simplified reference tissue method, with the cerebellar cortex, excluding the vermis, as a reference region.^31, 32^ Voxel-wise *t*-maps comparing BP_ND_ during MIST 1 relative to MIST 2 were generated using residual paired *t*-tests with a threshold of *t*=3.76 equivalent to *P*=0.05 for a whole striatum search volume based on random field theory.^33^ An objective of this approach is to detect changes in BP_ND_ at the voxel level with no *a priori* anatomical hypothesis, hence circumventing some of the limitations of volume of interest (VOI) placement.^27^ Please see the Supplementary Information for a more detailed description on how the striatal search volume and the voxel-wise statistical threshold were defined.

### VOI analysis

Three VOIs were selected bilaterally on each individual's MRI, including limbic ventral striatum, associative striatum (pre-commissural dorsal putamen, pre-commissural dorsal caudate and the post-commissural caudate) and sensorimotor striatum (post-commissural putamen). VOI delineation into gross anatomical brain structures was first obtained by applying automatic segmentation procedures to each individual's anatomical MRI.^34^ Each participant's set of VOI was then manually refined.^18^ To align the VOI template on PET dynamic data and extract regional time activity curves, each individual's dynamic radioactivity PET data were averaged along the time dimension and co-registered to the MRI.^35^ Estimates of average BP_ND_ within these VOIs were extracted in the three scanning conditions. A repeated-measures analysis of variance with experimental condition (MIST control, MIST 1, MIST 2) as within-subjects factor and one between-subjects factor subgroup (placebo, d-amphetamine) was conducted for each VOI, to investigate differences in BP_ND_. Degrees of freedom were corrected using the Greenhouse–Geisser test in the case of nonsphericity, as determined by the Mauchly test (see also Supplementary Information).

#### Subjective mood and Psychophysiology

Outcome measures were analyzed using repeated-measures analysis of variance. Subgroup (placebo vs d-amphetamine) was the between-subjects factor. Within-subjects factors for the behavioral data were experimental condition (MIST control, MIST 1, MIST 2) and time (baseline, post-task). For HR and cortisol, area under the curves (computed as in ref. 36) for each experimental condition was the within-subjects factor.

## Results

Eighteen healthy males took part in the study (d-amphetamine *n*=9; placebo *n*=9). One participant (d-amphetamine condition) displayed mean absolute changes in BP_ND_ (across regions) during exposure to MIST 1 (MIST 1 vs MIST control) three standard deviations above the sample mean (and five times higher than changes reported in response to ‘stress' in a previous study using the MIST).^27^ These abnormal BP_ND_ values in this participant were almost certainly due to a technical error. This participant was therefore removed from the analysis. The participants in the placebo vs d-amphetamine subgroups (Table 1) did not significantly differ with respect to demographics or personality measurements, or in injected dose of [^11^C]raclopride in any of the three PET sessions (see Table 1). Although injected amount appeared lower after stress 2 relative to stress 1, this effect was independent of the type of drug (d-amphetamine or placebo; *P*=0.94).

### PET study

#### Voxel-wise analyses

*Effect of stress exposure before repeated* *d*-*amphetamine or placebo (MIST 1 vs MIST control)*. Stress exposure before the d-amphetamine regimen (MIST 1 vs MIST control) elicited variable but significant decreases in striatal BP_ND_ values, primarily in the putamen. The magnitude of change (% decrease and cluster size) was quite similar for both subgroups (Table 2).

#### Effect of stress exposure following repeated d-amphetamine or placebo (MIST 2 vs MIST 1)

**Figure 2:**
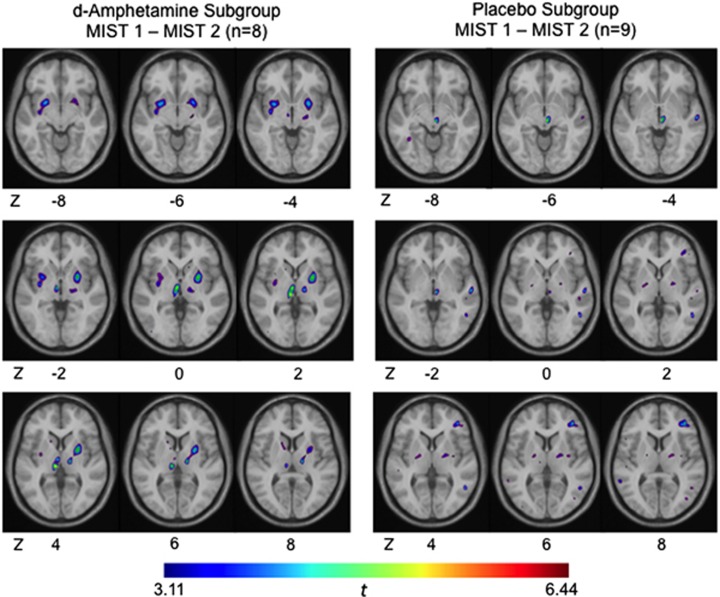
Voxel-wise *t*-maps of [^11^C]raclopride BP_ND_ changes during MIST who received the repeated d-amphetamine regimens (left, *n*=8) and placebo regimens (right, *n*=9), relative to the control condition. MIST 1–MIST 2=change in [^11^C]raclopride BP_ND_ during second exposure to stress relative to first stress exposure. A greater *t*-value reflects greater decreases in [^11^C]raclopride BP_ND_ (that is, greater dopamine release). BP_ND_, non-displaceable binding potential; MIST, Montreal Imaging Stress Task.

Although stress exposure before the d-amphetamine sensitization regimen elicited only small clusters of significantly decreased [^11^C]raclopride BP_ND_ values (see above), stress-induced decreases in BP_ND_ following the sensitizing regimen were much more widespread (Figure 2; Table 2 and Table 3). These larger clusters of decreased BP_ND_ following re-exposure to stress were not observed after the placebo regimen.

#### VOI analyses

VOI analyses showed that prior exposure to the d-amphetamine regimen led to highly variable, but nonsignificant, changes in [^11^C]raclopride BP_ND_, nor were there significant differences between MIST 2 and MIST 1 in the placebo group (Supplementary Figure S1). However, in the MIST 2 vs MIST control condition, secondary VOI analyses showed that, in the placebo group, significant decreases in BP_ND_ were observed in the right associative striatum (F(2,16)=4.44, *P*=0.03), left ventral striatum (F(2,16)=4.11, *P*=0.04) and right (F(2,16)=3.76, *P*=0.05) and left (F(2,16)=4.94, *P*=0.02) sensorimotor striatum.

### Behavior and psychophysiology

#### Mood states

Relative to MIST control, the MIST 1 stress exposure resulted in an increased ‘anxiety' response, as measured by the Profile of Mood States (experimental condition × time: F(2,30)=4.31, *P*=0.02; MIST 1 vs control: (1,15)=8.81; *P*=0.01) and the State-Trait Anxiety Inventory (experimental condition × time: F(2,30)=4.12, *P*=0.02; F(1,15)=8.41; *P*=0.01). These effects were not observed on MIST re-exposure at 21 days; nor did these effects differ between subgroups (d-amphetamine or placebo). There was no significant correlation between changes in [^11^C]raclopride BP_ND_ and the behavioral stress response. See also Supplementary Table S1.

#### Physiological measures

The MIST significantly increased heart rate during the first MIST PET scan and re-exposure at 21 days (main effect of experimental condition: F(2,30)=18.58, *P*<0.001; MIST 1 vs control: F(1,15)=19.66, *P*<0.001; MIST 2 vs control: F(1,15)=19.81; *P*<0.001), but there were no interactions with subgroup (amphetamine, placebo) or differences between MIST 1 vs MIST 2. Cortisol marginally increased during the MIST 1 exposure (F(1,15)=2.93; *P*=0.107) and more robustly at MIST re-exposure (at day 21; F(1,15)=18.88; *P*=0.001). The condition × subgroup interaction showed a trend towards significance (F(2,30)=3.15, *P*=0.057), with the cortisol response during re-exposure to the MIST greater following the d-amphetamine regimen (F(1,15)=5.20; *P*=0.038), relative to placebo. There were no significant correlations between changes in [^11^C]raclopride BP_ND_ and the psychophysiological or cortisol stress responses. Please see Supplementary Table S2.

#### Amphetamine levels

Consistent with our previous study,^18^ plasma amphetamine concentrations confirmed the presence of the stimulant drug in all the three sessions equally (see Supplementary Information for more details).

## Discussion

The present study investigated whether a regimen of d-amphetamine exposure previously demonstrated to induce sensitization in human volunteers would lead to greater responses to psychosocial stress. The results of the present study provide preliminary evidence that it might. Consistent with the hypothesis, the stress-induced DA and hypothalamic–pituitary–adrenal axis responses were significantly greater 14 days after a repeated d-amphetamine regimen. These heightened responses appear to be in line with reports of cross-sensitization in laboratory animals.^8, 11, 37^

DA release in the nucleus accumbens has been well documented in experimental animals following exposure to stressful events, such as electric shock, tail pinch and bodily restraint.^38, 39, 40^ In humans, few studies have investigated dopaminergic responses to stress. The DA responses to psychosocial stress appear highly variable and are often limited to susceptible individuals (for example, individuals with low self-esteem, a history of low maternal care or those at risk for psychosis)^26, 27, 41^ The present study raises the possibility that these variable responses might reflect, in part, differential lifetime histories of stressful experiences.

Consistent with the main hypothesis here, it was found that, following repeated d-amphetamine, re-exposure to stress further decreased BP_ND_ values in the healthy participants. These findings are reminiscent of observations of altered [^11^C]-(+)-PHNO (a D_2_/D_3_ agonist ligand) binding responses in individuals with psychosis, using this laboratory stress paradigm.^42^ The present findings strengthen the view that repeated exposure to drugs, over and above other vulnerability factors (for example, genetic), might summate their specific effects to alter stress responses in striatal areas, and, possibly, the risk of DA-related disorders.

Changes in BP_ND_ were also observed following the placebo regimen. The reported changes in the d-amphetamine subgroup were regionally specific, occurring in the left ventral striatum and bilaterally in the posterior putamen. Repeated stress alone has been demonstrated to alter meso-corticolimbic DA release in animal models.^43^ In humans, previous stress exposure, particularly early-life stress, has been identified as one important factor for the development of psychiatric disorders later in life.^44, 45^ Although it is currently impossible to establish a direct causal relationship, it has been demonstrated that early-life stress is associated with increased ventral striatum DA release to subsequent stress^26^ as well as to psychostimulant exposure later in life.^46^ Our findings of decreased BP_ND_ (in the placebo subgroup) supports previous literature that repeated, uncontrolled stress exposure alone, can lead to sensitization.^3^ Re-exposure to stress following d-amphetamine (relative to placebo) may produce different effects in different striatal subregions.^18^

Although the results are in line with studies showing increased stress or amphetamine-induced DA responses in laboratory animals that had been previously exposed to repeated amphetamine,^3^ the results contrast with evidence of attenuated DA responses in patients with substance use disorders (relative to controls) following an acute challenge with methylphenidate or amphetamine^47, 48, 49^ or exposure to a laboratory stressor.^41^ The reasons for this discrepancy remain unclear, but could reflect pre-existing traits, protracted withdrawal effects in those with extensive histories of substance abuse or a shift from DA to other neurobiological substrates mediating heightened behavioral responses to various challenges.^50, 51, 52^ Together, these findings highlight the need to study systematically DA (cross) sensitization mechanisms in clinical samples with various levels of prior drug exposure to further understand the relevance of DA (cross)sensitization in the onset and relapse of drug dependence/abuse.

### Strengths and limitations

This study benefitted from selecting a carefully screened, homogenous sample of males who were all monitored carefully for drug use and stressful experiences throughout the 30-day period of testing, thus minimizing potential confounds. It would be of interest, however, to determine whether the results could be generalized to other samples, including females, patients or following the exposure to chronic repeated stress. Longitudinal follow-up here would help.

Though this sample size was no different from the one examined in previous sensitization studies (including our own), it did not allow reliable investigation of higher-order interactions between personality, psychophysiology and DA response. A larger sample would have also allowed to study the potential moderating role of specific alleles/genotypes (for example, Met allele of the COMT Val(158)Met polymorphism, such as in Hernaus *et al.*^53^). Similarly, our sample may not have provided sufficient statistical power to detect significant correlations between BP_ND_ and behavioral and physiological measures. Moreover, the VOI analyses indicated that the DA response to stress re-exposure after the d-amphetamine regimen was highly variable. This high variability is hypothesized to account for the fact that the observed effects using *t*-maps could not be confirmed by VOI analyses. Alternatively, the activation loci in the *t*-maps differed from the VOI boundaries and thus may not have been revealed by VOI analyses. Indeed, striatal subregions based on cortical functional connectivity in humans appear to be larger in number than suggested by the tripartite model.^54^

Though the initial stress exposures elicited the expected effects on HR and negative mood states,^55^ re-exposure to stress after subchronic d-amphetamine administration did not elicit negative mood. The absence of mood lowering on stress re-exposure may be explained by the fact that behavioral sensitization to psychostimulants in healthy individuals may mainly be expressed as mood-elevation, arousal or psychomotor effects^18^ alterations that may even counteract negative responses to psychosocial stress.

The increased DA response to stress in the subchronic d-amphetamine subgroup might have been influenced by testing participants in the environment that had been paired with the drug. For example, in laboratory animals, drug-paired stimuli can facilitate the expression of DA sensitization and elicit long-lasting conditioned DA release.^56, 57^ Our previous studies have identified evidence of these same effects in humans. When the participants were tested in the drug-paired PET environment, we obtained evidence of drug-induced DA sensitization^18^ and conditioned DA release.^58^ In comparison, re-exposure to the drug-paired environment in the absence of the discrete drug cue (placebo capsule) did not lead to a conditioned DA response. As, in the present study, our final stress challenge was given in the drug-paired environment without a placebo capsule, it is possible that the expression of cross-sensitization was augmented by the drug-paired stimuli, while reflecting something other than the adding together of conditioned and stress-induced DA responses.^59, 60^

Although a strength of the study was the use of a well-validated method, [^11^C]raclopride is only sensitive to changes in DA release in the striatum. It would be of interest to study whether cross-sensitization or sensitization to stress also occurs in extrastriatal areas (for example, using [^18^F]fallypride). Indeed, we previously demonstrated DA release in the dorsal medial prefrontal cortex following acute laboratory psychosocial stress.^55^

Another potential limitation is the controllable nature of the stress exposure. Pre-clinical research has distinguished between two specific classes of stressors in their ability to precipitate sensitization: ‘controllable' versus ‘uncontrollable' events.^61^ Uncontrollable, intermittent stress appears to be a key feature of stressful events that trigger the neurobiological changes leading to cross-sensitization.^62, 63^ For ethical reasons, participants were allowed to terminate the experiment at any time they would choose, which may have reduced the perceived uncontrollability of the situation, allowing for a degree of ‘control' which could have influenced stress ‘responses', thus ‘sensitization' to stress.

Finally, although the participants were carefully screened for use of drugs in the past and a urine drug test was done at the beginning of each session, some participants had used tobacco or cannabis in the past, and the timing of last nicotine exposure was not confirmed by blood testing. On the basis of the findings, in animals, that repeated nicotine or cannabis exposure can induce sensitization,^64^ it could be argued that participants who had previously smoked may already be sensitized, hence the theoretical possibility of another confound. However, the amount of prior nicotine or cannabis exposure was very low. Moreover, the d-amphetamine and placebo group did not significantly differ in their prior use, and an effect of the stress-amphetamine regimen could still be observed despite the potential influence of past use.

## Summary and conclusion

The present study provides preliminary experimental evidence *in vivo* that DA sensitization to psychostimulants may generalize to stress in humans. Sensitization-like phenomena have been frequently proposed to account for stress-induced relapses in addiction or psychosis, that is, in disorders in which DA is believed to have a major role.^2, 65^ The present study tentatively identified amphetamine-related effects that could potentially be linked to how repeated drug exposure would progressively lead to onset or relapse, particularly for putative DA-related disorders, when someone is exposed to further life stressors.

Interestingly, repeated stress exposure alone also elicited some DA release within the striatum. Nevertheless, though speculative, it offers some support for the theory that repeated stressors with or without stimulants, may trigger a cascade of neurobiological events^66^ that may also influence the onset of or relapse to a number of DA-related disorders. In particular, the specific role of ‘sensitization' to repeated ‘stress' was envisaged, highlighted and discussed.

Previous studies have raised the possibility that sensitization and cross-sensitization could be relevant for the development and expression of psychiatric phenomenology in vulnerable individuals.^4, 5, 6, 7, 67^ For example, in a study of cocaine users who had experienced drug-induced psychotic reactions, 65% reported becoming progressively more susceptible to these effects (that is*,* paranoid ideation became more severe or was triggered by lower doses, indicative of behavioral sensitization), and these individuals were more likely to relapse to drug use at follow-up, as indexed by a greater number of re-hospitalizations.^5^ Determining more definitively whether DA sensitization underlies increased susceptibility to these and other problems will require longitudinal behavioral, neuroimaging and psychopharmacological challenge studies. Moreover, it will be important to determine the relevance of sensitization and cross-sensitization to a nonspecific lowering of the threshold for diverse symptoms vs specific symptoms; for example*,* autonomic system responsiveness, acute anxiety reactions, psychotic ‘breaks', manic symptoms including increased goal-directed behaviors and renewed bouts of drug seeking and use. Although the proposition that the present observations can be generalized to psychiatric samples is compelling, for now, this remains to be confirmed.

## Figures and Tables

**Table 1:** Characteristics of the sample (mean (s.d.))

*Variable*	*d-Amphetamine subgroup (*n=*8)*	*Placebo subgroup (*n=*9)*	P-*value*
Age	22.8 (4.6)	21.5 (1.9)	0.45
Beck Depression Inventory	1.9 (2.2)	1.5 (2.3)	0.78
Cigarettes per day^a^	0.4 (0.7)	0.02 (0.07)	0.18
No. of drinks a week	4.1 (3.3)	2.4 (2.5)	0.25
No. of times cannabis used in last 30 days^b^	0.4 (0.5)	0.1 (0.3)	0.22
% University students	100%	100%^c^	NA
Spielberger Trait Anxiety Scale	31.1 (9.2)	30.1 (7.9)	0.80
Rosenberg Scale	24.2 (4.4)	25.0 (4.0)	0.71

*Questionnaire of competence and control*			
Self-esteem	35.6 (3.5)	33.8 (4.7)	0.40
Internality	33.6 (7.2)	32.5 (3.1)	0.69
Perceived control by others	24.9 (3.6)	24.0 (6.3)	0.73
Chance	19.9 (6.8)	21.4 (4.0)	0.56
			
*Injected [^11^C]raclopride dose (mCI)*			
Control	7.12 (0.3)	7.01 (0.8)	0.70
Stress 1	7.01 (0.3)	7.24 (0.3)	0.12
Stress 2	6.66 (0.4)	6.88 (0.3)	0.25

Abbreviation: NA, not available.

a Two participants were light smokers (AMPH condition: one on average two cigarettes per day; one on average one cigarette a day; placebo condition: one of average one a week).

b Three participants reported to have used cannabis in the past 30 days on one occasion in AMPH condition. One participant in the placebo condition reported to have used cannabis in the past 30 days on one occasion before his first session.

c Education level was missing for one participant in the placebo group.

**Table 2:** Illustration of included BP_ND_ data (±s.d.) from the largest cluster in each *t*-map

	*MIST 1*–*MIST control cluster*	*MIST 2*–*MIST control cluster*	*MIST 2*–*MIST 1 cluster*
*d-Amphetamine subgroup*			
BP_ND_			
MIST control	**1.5±0.64**	**2.18±0.39**	2.44±0.47
MIST 1	**1.28±0.57**	2.09±0.42	**2.43±0.42**
MIST 2	1.33±0.78	**1.92±0.45**	**2.21±0.45**

Delta BP_ND_			
MIST 1–MIST control	**−14.95±14.24**	−4.18±9.28	0.49±11.36
MIST 2–MIST control	−15.37±28.53	**−10.1±20.85**	−7.27±22.49
MIST 2–MIST 1	−0.82±31.82	−5.72±23.17	**−8±17.87**

*Placebo subgroup*			
BP_ND_			
MIST control	**1.5±0.27**	**2.15±0.24**	1.85±0.28
MIST 1	**1.29±0.21**	2.01±0.24	**1.95±0.17**
MIST 2	1.44±0.33	**1.92±0.17**	**1.73±0.21**

Delta BP_ND_			
MIST 1–MIST control	**−12.53±12.62**	−6.61±6.41	6.79±13.9
MIST 2–MIST control	−4.1±13.19	**−10.48±6.07**	−5.86±9.82
MIST 2–MIST 1	11.88±22.98	−3.78±8.71	**−11.35±7.5**

Abbreviations: BP_ND_, non-displaceable binding potential; MIST, Montreal Imaging Stress Task.

As the clusters were identified on the basis of their significance, the BP_ND_ values extracted from those clusters are by definition statistically different. Those values that were used to generate the *t*-map are in bold.

**Table 3:** Detailed information regarding the identified clusters and coordinates of the peak voxel (Montreal Neurological Institute space coordinates) within those clusters

*Comparison*	*Cluster*	*Volume (mm*^*3*^)	*Resels*	P-*value*	*x*	*y*	*z*
d-*Amphetamine subgroup*							
MIST 1–MIST control							
	1	288	4.5	<0.001	−9	16	9
	2	111	1.73	0.004	33	−11	4
	3	57	0.89	0.047	27	12	4
							
	MIST 2–MIST control							
	1	4997	78.08	<0.001	29	10	1	
	2	3691	57.67	<0.001	−14	14	5	
	3	405	6.33	<0.001	−26	−10	7	
							
MIST 2–MIST 1								
	1	2455	38.36	<0.001	26	4	1	
	2	1695	26.48	<0.001	−23	6	−8	
	3	561	8.77	<0.001	−15	3	13	
	4	105	1.64	0.005	17	13	12	
								
*Placebo subgroup*								
MIST 1–MIST control								
	1	507	7.92	<0.001	16	21	−4	
	2	312	4.88	<0.001	−25	15	−8	
	3	263	4.11	<0.001	28	−13	−6	
	4	98	1.53	0.006	−25	−3	−6	
						
MIST 2–MIST control								
	1	3935	61.48	<0.001	−23	12	−9	
	2	1998	31.22	<0.001	31	−12	2	
								
	MIST 2–MIST 1							
	1	125	1.95	0.002	−16	−2	16	
	2	87	1.36	0.011	23	−6	5	
	3	52	0.81	0.062	−24	−10	3	

Abbreviation: MIST, Montreal Imaging Stress Task.
