# Supplementary material for: Dopamine cross-sensitization between psychostimulant drugs and stress in healthy male volunteers
Source: Transl Psychiatry. 2016 Feb 23;6(2):e740–. doi: 10.1038/tp.2016.6 (PMC4872435; doi:10.1038/tp.2016.6)
Supplement: Supplementary Information [file tp20166x1.docx]

**SUPPLEMENTARY SECTION**

**METHODS**

**Participants**

All participants scored one or more standard deviations (SD) above the normal population mean on the novelty-seeking (NS) scale of Cloninger’s Tridimensional Personality Questionnaire (TPQ).[^1^](#_ENREF_1) The choice to select subjects with high TPQ NS scores is based on observations that NS scores in humans predict [^11^C]raclopride binding potential (*BP*_ND_) responses to single and repeated doses of *d*-amphetamine [^2^](#_ENREF_2) [^3^](#_ENREF_3) and amphetamine-induced behavioural sensitization. Though animal studies raise the possibility that women might be more sensitive to sensitization effects than men [^4^](#_ENREF_4), we selected only males to rule out effects of estradiol-induced variability in psychophysiological response to stress throughout the menstrual cycle.[^5^](#_ENREF_5)

**PET and magnetic resonance imaging acquisition protocols**

Participants underwent three 60-minute dynamic PET scans, performed on a CTI/Siemens (Knoxville, TN) ECAT-HR+ PET camera with lead septa removed (63 slice coverage, with a maximum resolution of 4.2 mm full-width half-maximum (FWHM) in the centre of the field of view). Ten-minute transmission scans for attenuation correction were performed using a ^68^Ga source prior to tracer injection. A catheter was inserted into the subject’s antecubital vein for 7 mCi bolus tracer injection (mean ± SD: 7.0 ± 0.5) and blood drawing. Emission data were collected across 60 minutes in 26 time frames of progressively longer duration. For the purpose of PET/MRI co-registration, all participants also underwent high-resolution magnetic resonance imaging (MRI) using a 1.5-T superconducting magnet system (Sonata; Siemens) using gradient echo pulse sequence (repetition time = 9.7 msec, echo time = 4 msec, flip angle = 12°, field of view = 250 and matrix 256 x 256).

### **Pill sessions**

Following the first two PET scans, subjects received *d*-amphetamine (0.3mg/kg) or placebo, on three separate days within 7 days. The *d*-amphetamine dose was chosen based on studies showing its efficacy at inducing behavioral and dopamine sensitization. [^2^](#_ENREF_2) Behavioral and physiological measures (POMS, Spielberger, VAS, HR, cortisol) were taken at regular time intervals throughout the sessions (baseline, 1h after d-amphetamine, 60, 90 and 120 mins after d-amphetamine). The *d*-amphetamine and placebo sessions took place within the same test environment as the actual PET scans (subjects were lying on the PET camera bed), as it was expected that this would facilitate the expression of sensitization.[^6^](#_ENREF_6)^,^ [^7^](#_ENREF_7) Plasma amphetamine concentrations were analyzed using electron-capture gas chromatography after extraction and derivatization of amphetamine according to a standardized protocol.[^8^](#_ENREF_8) Administration led to the expected plasma concentrations (mean peak amphetamine plasma levels during the 3^rd^ dose: ± SD: 30.4± 20.4 ng/ml). No amphetamine could be detected on the days of the PET sessions.

### **Voxel-wise parametric map and t-statisitcs**

**EXPERIMENTAL DESIGN**

**PLACEBO**

^11^ C-scan

Day

**≥ 21**

≥ 14-day

Latency

…

**AMPH**

**AMPH**

**AMPH**

No drug

*Sham* scan

*Sham* scan

^11^ C-scan

^11^ C-scan

Day

**5**

Day

**3**

Day

**1**

Day

***0**

PET images were reconstructed using a 6-mm FWHM Hanning filter and corrected for movement by applying an algorithm that compensates for between-frame misalignment.[^9^](#_ENREF_9) [^11^C]Raclopride time activity curves were obtained from the dynamic data and specific binding (BP_ND_) was estimated at each voxel using the simplified reference tissue method (SRTM)[^10^](#_ENREF_10)^,^ [^11^](#_ENREF_11) and the time activity curve of the cerebellar cortex excluding vermis as a reference tissue devoid of DA D_2/3_ receptors. Parametric images of [^11^C]raclopride BP_ND_ were spatially normalized to an anatomical template (see below PET MRI coregistration) and investigated for changes in [^11^C]raclopride BP_ND_ between conditions (MIST stress and MIST control) by applying a t-statistical test at every voxel.[^12^](#_ENREF_12) Paired t-tests were computed to estimate significant change in [^11^C]raclopride BP_ND_ associated with exposure to stress relative to control before (MIST control – MIST at day 1). The question of sensitization (i.e., greater stress-induced changes in [^11^C]raclopride BP_ND_ after repeated amphetamine regimen) was addressed by investigating the contrast between MIST, day 1 vs MIST, day 21 in the *d*-amphetamine and placebo groups separately. Voxels with a t ≥ 3.76 were considered significant at p ≤ .05, Bonferroni corrected for multiple comparisons.[^13^](#_ENREF_13)

**Defining Striatal Search Volume**

The striatal search volume was delineated on each individual's MRI through a semi-automated non-linear transformation between a high-resolution model and the individual's brain. Each striatal label was manually verified and corrected. The striatal search volume was established by summing the striatal mask images of all subjects in stereotaxic space, and then selecting voxels as part of the mask that were identified as striatal in at least ½ of the participants. This average striatal volume was then expanded by 1 mm in all directions.

**Determination of voxel-wise statistical threshold**

Pairwise comparisons of BP_ND_ maps were made using a voxelwise t-test calculated using BP_ND_ values and the standard deviations of BP_ND_, calculated using the residuals of the fitted compartmental model (See ^[12](#_ENREF_12)^). The statistical thresholds were then calculated for both individual voxels and peaks using a publicly available Matlab script (<http://www.math.mcgill.ca/keith/fmristat/toolbox/stat_threshold.m>). The parameters used were a search volume of 39293 mm^3^ = 4911.6 2 mm^3^ voxels, a full width half maximum of the images of 4 mm, and 368 or 414 degrees of freedom. The degrees of freedom were calculated as df = 2×N×(k-p) where N=number of subjects in the t-map (8 for Amphetamine subgroup, 9 for controls), k=frames in dynamic pet (26) and p=the number of parameters in the simplified reference tissue model (3: R1, K2, and BP_ND_). This yields a single voxel threshold of 4.3176 for the subjects in the amphetamine condition and 4.3115 for the subjects in the placebo condition. Cluster thresholds were 3.1130 for the subjects in the amphetamine condition and 3.1105 for the subjects in the placebo condition, with minimum cluster extent thresholds of 56.00 mm and 56.01 mm. The calculation of the single voxel thresholds is based on the method proposed by [^13^](#_ENREF_13), while cluster thresholds are calculated using the method of Cao [^14^](#_ENREF_14). The T-map images were thresholded at a minimum t of 3.76 for visualization purposes.

**Region of Interest Analysis**

ROI delineation and analyses were performed by using individual anatomical MRI. MRI volumes were corrected for image intensity non uniformity[^15^](#_ENREF_15) and linearly and nonlinearly transformed into standardized stereotaxic space[^16^](#_ENREF_16) using automated feature matching to the MNI305 template.[^17^](#_ENREF_17) Three regions of interest (ROIs) were selected bilaterally on each individual’s MRI using a combination of manual and automatic approaches. ROI delineation into gross anatomical brain structures was first obtained by applying a tissue classification (gray matter, white matter, and CSF) and automatic segmentation.[^18^](#_ENREF_18) Each subject’s set of ROI was then manually refined as described elsewhere.[^19^](#_ENREF_19) In order to align ROI template on PET dynamic data and extract regional time activity curves each individual’s dynamic radioactivity PET data was averaged along the time dimension and coregistered to the MRI. [^20^](#_ENREF_20) [^11^C]Raclopride time activity curves were extracted from each ROI and BP_ND_ was estimated using SRTM. A repeated measures MANOVA with condition as within subjects factor and one between subjects factor (Group: placebo and d-Amph) for each of the ROIs was conducted to investigate the difference between ROI [^11^C]raclopride BP_ND_ across Stress Conditions (MIST control, MIST 1, MIST 2) and Group (placebo vs d-Amph). Degrees of freedom were corrected using the Greenhouse Geisser in case of non-sphericity, as determined by the Mauchly test. When appropriate, Least Significant Difference t-tests were applied to determine the significance of change in BP_ND_ across conditions.

**RESULTS**


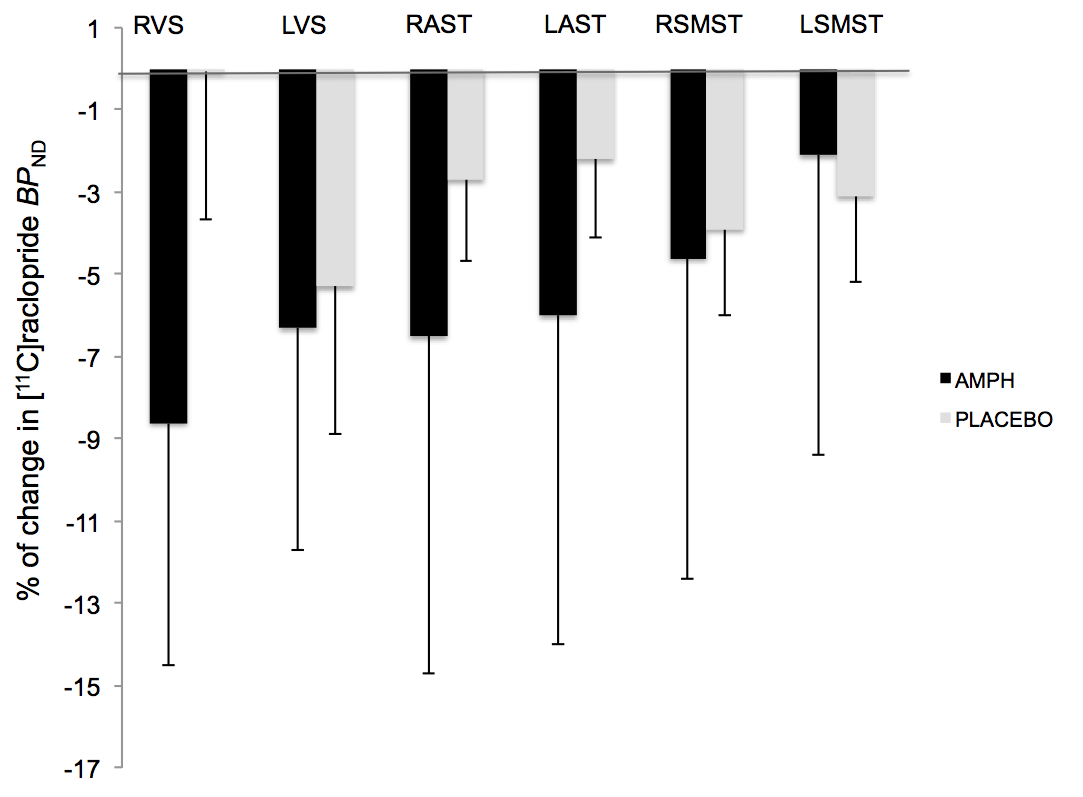


**Figure S1**. Percentage of change in [^11^C]raclopride *BP*_ND_ values (mean ± SE); MIST 1-MIST 2 = change in [^11^C]raclopride *BP*_ND_ during second exposure to stress relative to first stress exposure. A lower value reflects a greater decrease in [^11^C]raclopride *BP*_ND_ (i.e., greater DA release). RVS=right ventral Striatum; LVS=left ventral striatum; RAST=right associative striatum; LAST=left associative striatum; RMST=right sensorimotor striatum; LMST=left sensorimotor striatum.

**Table S1.** Mean (+/- SE) scores on the POMS anxiety scale and STAI, as a function of subgroup (d-amphetamine vs. placebo) and experimental condition (control, MIST 1, MIST 2). A lower score on the POMS reflects more anxiety. A higher score on the STAI reflects more anxiety.

| ***d*-AMPHETAMINE SUBGROUP** | | | | | | |
| --- | --- | --- | --- | --- | --- | --- |
|  | ***CONTROL*** | | ***MIST 1*** | | ***MIST 2*** | |
|  | Before MIST | After MIST | Before MIST | After MIST | Before MIST | After MIST |
| POMS anxiety | 58(3) | 56.1(3.4) | 57.2(2.8) | 49.1(4)^1^ | 58.6(3.5) | 53.9(3.2) |
| STAI | 29.9(2.6) | 35.5(2.7) | 31(2.8) | 43.6(3.4)^2^ | 30.2(3.7) | 36.9(2.6) |
| **PLACEBO SUBGROUP** | | | | | | |
|  | ***CONTROL*** | | ***MIST 1*** | | ***MIST 2*** | |
|  | Before MIST | After MIST | Before MIST | After MIST | Before MIST | After MIST |
| POMS anxiety | 55.3 (3.2) | 56.0 (3.5) | 56 (2.8) | 52.7(3.3)^1^ | 53.6(3.8) | 53.3(2.5) |
| STAI | 32.4(2.9) | 36.6(4.2) | 32.9(2.9) | 41.6(3.1)^2^ | 37.3(2.9) | 40.78(2.9) |

^1^ Experimental condition X time: F(2,30)= 4.31, p=0.02; MIST 1 vs. control: (1,15)=8.81; p=0.01. There were no interactions with subgroup.

^2^ Experimental condition X time: F(2,30)= 4.12, p=0.02; F(1,15)=8.41; p=0.01. There were no interactions with subgroup.

**Table S2.** AUC (+/- SE) in Heart rate and cortisol, as a function of subgroup (d-amphetamine vs. placebo) and experimental condition (control, MIST 1, MIST 2).

|  | Control | MIST 1 | MIST 2 |
| --- | --- | --- | --- |
| ***d*-AMPHETAMINE SUBGROUP** | | | |
| Heart rate (AUC) | 289 (15) | 344(21)^1^ | 364(17) ^1^ |
| Cortisol (AUC) | 1138(185) | 1153(164)^2^ | 1616(204) ^2^ |
| **PLACEBO GROUP** | | | |
| Heart rate (AUC) | 314(20) | 343(22)^1^ | 357(23) ^1^ |
| Cortisol (AUC) | 1076(101) | 1439(102) ^2^ | 1381 (129) ^2^ |

^1^ Main effect of experimental condition: F(2,30)= 18.58, p<0.001; MIST 1 *vs*. control: (1,15)=19.66, p<0.001; MIST 2 *vs*. control: F(1,15)=19.81; p<0.001). There were no interactions with subgroup (amphetamine, placebo), or differences between MIST 1 *vs*. MIST 2.

^2^ Relative to control, Cortisol marginally increased during the MIST 1 exposure (F(1,15)=2.93; p=0.107) and more robustly at MIST re-exposure (at day 21) (F(1,15)=18.88; p=0.001). The Condition x Subgroup interaction showed a trend towards significance (F(2,30)=3.15, p=0.057), with the cortisol response during re-exposure to the MIST greater following the *d-*amphetamine regimen (F(1,15)=5.20; p=0.038), relative to placebo

**Analyses of Amphetamine levels in blood**

Plasma amphetamine concentrations increased in all three sessions equally. Specifically, in the amphetamine group, plasma amphetamine concentrations increased in all three sessions equally (main effect of time: F(3,21) = 31.42, p<.001; main effect of day: F(2,14)=0.49, p=.62; day X time interaction: F(6,42), =0.65, p=.69). There were no significant correlations between changes in [^11^C]raclopride *BP*_ND_ and the maximum increase in amphetamine levels. Plasma amphetamine levels for the placebo subgroup were not analyzed.

**References**

1. Cloninger CR, Przybeck TR, Svrakic DM. The Tridimensional Personality Questionnaire: U.S. normative data. *Psychol Rep* 1991; **69:** 1047-1057

2. Boileau I, Dagher A, Leyton M, Gunn RN, Baker GB, Diksic M*, et al*. Modeling sensitization to stimulants in humans: an [11C]raclopride/positron emission tomography study in healthy men. *Archives of general psychiatry* 2006; **63:** 1386-1395.

3. Leyton M, Boileau I, Benkelfat C, Diksic M, Baker G, Dagher A. Amphetamine-induced increases in extracellular dopamine, drug wanting, and novelty seeking: a PET/[11C]raclopride study in healthy men. *Neuropsychopharmacology* 2002; **27:** 1027-1035.

4. Strakowski SM, Sax KW, Setters MJ, Keck PE, Jr. Enhanced response to repeated d-amphetamine challenge: evidence for behavioral sensitization in humans. *Biological psychiatry* 1996; **40:** 872-880.

5. Kirschbaum C, Kudielka BM, Gaab J, Schommer NC, Hellhammer DH. Impact of gender, menstrual cycle phase, and oral contraceptives on the activity of the hypothalamus-pituitary-adrenal axis. *Psychosom Med* 1999; **61:** 154-162.

6. Leyton M. Conditioned and sensitized responses to stimulant drugs in humans. *Progress in neuro-psychopharmacology & biological psychiatry* 2007; **31:** 1601-1613.

7. Vezina P, Leyton M. Conditioned cues and the expression of stimulant sensitization in animals and humans. *Neuropharmacology* 2009; **56 Suppl 1:** 160-168.

8. Asghar SJ, Baker GB, Rauw GA, Silverstone PH. A rapid method of determining amphetamine in plasma samples using pentafluorobenzenesulfonyl chloride and electron-capture gas chromatography. *Journal of pharmacological and toxicological methods* 2001; **46:** 111-115.

9. Costes N, Dagher A, Larcher K, Evans AC, Collins DL, Reilhac A. Motion correction of multi-frame PET data in neuroreceptor mapping: simulation based validation. *Neuroimage* 2009; **47:** 1496-1505.

10. Gunn RN, Lammertsma AA, Hume SP, Cunningham VJ. Parametric imaging of ligand-receptor binding in PET using a simplified reference region model. *Neuroimage* 1997; **6:** 279-287.

11. Lammertsma AA, Hume SP. Simplified reference tissue model for PET receptor studies. *Neuroimage* 1996; **4:** 153-158.

12. Aston JA, Gunn RN, Worsley KJ, Ma Y, Evans AC, Dagher A. A statistical method for the analysis of positron emission tomography neuroreceptor ligand data. *Neuroimage* 2000; **12:** 245-256.

13. Worsley KJ, Marrett S, Neelin P, Vandal AC, Friston KJ, Evans AC. A unified statistical approach for determining significant signals in images of cerebral activation. *Human brain mapping* 1996; **4:** 58-73.

14. Cao J. The Size of the Connected Components of Excursion Sets of χ 2, t and F Fields. *Advances in Applied Probability* 1999; **31:** 579-595.

15. Sled J. A non-parametric method for automatic correction of intensity non-uniformity in MRI data. *IEEE Trans Med Imaging* 1997.

16. Talairach J, Tournoux P. *Co-planar stereotaxic atlas of the human brain*. Thieme: New York: 1988.

17. Collins DL, Neelin P, Peters TM, Evans AC. Automatic 3D intersubject registration of MR volumetric data in standardized Talairach space. *J Comput Assist Tomogr* 1994; **18:** 192-205.

18. Collins D, Evans A, Holmes C, Peters TM. *Automatic 3D segmentation of neuro-anatomical structures from MRI*. Kluwer Academic Publishers: 1995.

19. Martinez D, Slifstein M, Broft A, Mawlawi O, Hwang DR, Huang Y*, et al*. Imaging human mesolimbic dopamine transmission with positron emission tomography. Part II: amphetamine-induced dopamine release in the functional subdivisions of the striatum. *Journal of cerebral blood flow and metabolism* 2003; **23:** 285-300.

20. Evans AC, Marrett S, Neelin P, Collins L, Worsley K, Dai W*, et al*. Anatomical mapping of functional activation in stereotactic coordinate space. *Neuroimage* 1992; **1:** 43-53.
